# Prognostic significance of artemin and GFRα1 expression in laryngeal squamous cell carcinoma

**DOI:** 10.3892/etm.2014.1821

**Published:** 2014-07-02

**Authors:** CHAOBING GAO, XINGWANG CHENG, XIAOHONG LI, BUSHENG TONG, KAILE WU, YEHAI LIU

**Affiliations:** 1 Department of Otorhinolaryngology, Head and Neck Surgery, The First Affiliated Hospital of Anhui Medical University, Hefei, Anhui 230022, P.R. China; 2 Department of Emergency, The First Affiliated Hospital of Bengbu Medical University, Bengbu, Anhui 233030, P.R. China

**Keywords:** artemin, GFRα1, laryngeal squamous cell, survival

## Abstract

Artemin (ARTN) has been implicated in the development and progression of several human malignancies. However, the clinical and prognostic significance of ARTN and its receptors has not yet been investigated in human laryngeal squamous cell carcinoma (LSCC). Therefore, in the present study, the protein expression of ARTN and its receptor, namely GFRα1, was determined in 76 LSCC and 26 laryngeal polyp tissue samples using immunohistochemistry. Furthermore, the clinicopathological and prognostic significance of ARTN and GFRα1 expression was analyzed in patients with LSCC. The results revealed that the expression of ARTN and GFRα1 was significantly increased in LSCC compared with polyp tissue samples. Furthermore, the expression of ARTN and GFRα1 was positively associated with pTNM stage in LSCC. Kaplan-Meier survival analyses revealed a strong association between the expression of ARTN or GFRα1 and the survival of patients with LSCC. Correlation analysis demonstrated that the expression of ARTN was significantly correlated with the expression GFRα1. In conclusion, the results demonstrated that ARTN and GFRα1 may be useful predictors of disease progression and outcome in patients with LSCC.

## Introduction

Laryngeal squamous cell carcinoma (LSCC) is one of the most common malignancies in the head and neck region, which leads to 350,000 mortalities worldwide each year (1,2). Despite considerable advances in diagnosis and therapy, the survival of patients with LSCC remains poor. At present, there are no effective biomarkers for LSCC to assist early diagnosis or monitor patient prognosis. Furthermore, traditional prognostic markers, including tumor histological grade, clinical stages and lymph node metastasis, may not fully evaluate the patient’s survival (3). Therefore, improving the understanding of the molecular mechanisms and gene alterations involved in the development and progression of LSCC may be helpful for the establishment of novel biomarkers to effectively monitor patients with LSCC.

Artemin (ARTN) is a growth factor that belongs to the glial cell line-derived neurotrophic factor (GDNF) family of ligands (GFL), which consists of four members, including GDNF, neurturin and persephin (4,5). GFL family members (including ARTN) have been found to signal via interaction with one or more of the GDNF receptor α family (GFRα), which is comprised of four members, GFRα1–4 (5–7). In addition to the role of neurotrophic factor (5–7), ARTN has also been found to have an oncogenic role in promoting tumor growth, migration, invasiveness and metastasis in a number of types of human cancer (8–14). For example, it has been demonstrated that the expression of ARTN is significantly increased in breast cancer tissues compared with normal breast tissues, and that high expression of ARTN is positively correlated with high tumor stage and poor survival in breast cancer (9). In endometrial cancer, high expression levels of ARTN have been observed be significantly associated with high tumor grade and myometrial invasiveness in clinical tissue specimens, and forced expression of ARTN has been demonstrated to increase tumor cell growth and invasiveness *in vivo* and *in vitro* (11). In addition, the expression levels of ARTN and its receptors have been observed to be upregulated in breast cancer and significantly associated with disease progression (15). Furthermore, co-expression of ARTN with its receptors has been found to produce synergistic increases in the odds ratio for survival in patients with breast cancer (15). These results suggest that ARTN with its receptors may have an important role in human solid tumors. However, to the best of our knowledge, the clinical impact and prognostic significance of ARTN or its receptor expression in human LSCC has not yet been investigated.

In the present study, the protein expression of ARTN and one of its receptors, GFRα1, in LSCC was determined using immunohistochemistry and the correlation between the expression levels, clinicopathological features and patient survival outcome was analyzed. The aim was to investigate whether ARTN and its receptors may be potential biomarkers of disease progression and prognosis in patients with LSCC.

## Materials and methods

### Patients and specimens

The patient population consisted of 76 consecutive patients with LSCC and 26 consecutive patients with benign polyp, who underwent surgery at the First Affiliated Hospital of Anhui Medical University (Hefei, China) between 2007 and 2009. None of the patients had undergone any chemotherapy or radiation therapy prior to the surgery, or had a previous diagnosis of carcinoma or a distant metastasis at the time of diagnosis. The pathohistological diagnosis and tumor histological grade of the patients was based on the World Health Organization (16). The pathological tumor staging (pstage) was determined according to the TNM classification of malignant tumors by the International Union Against Cancer (UICC, 2002) (17). The median time of patient follow up was 60 months. This study was approved by the institutional review board of the First Affiliated Hospital of Anhui Medical University (Anhui, China) and written, informed consent was obtained from all patients.

### Immunohistochemistry

Formalin-fixed, paraffin-embedded tissues were collected from each patient and cut into 4-μm-thick sections. Immunohistochemical analysis of ARTN and GFRα1 protein expression was performed using polyclonal antibodies against ARTN (1:100 dilution; R&D Systems, Minneapolis, MN, USA) and GFRα1 (1:100 dilution; Santa Cruz Biotechnologies, Santa Cruz, CA, USA) using the peroxidase-conjugated streptavidin complex method (Histostain-SP kit; Zymed, San Francisco, CA, USA), as previously described (18).

### Review and scoring

The results of the immunoreactivity of stained sections were reviewed and scored for expression of ARTN and GFRα1 using a light microscope (Olympus American Inc., Melville, NY, USA) by two pathologists in a blinded manner. The sections were scored based on the staining intensity and the percentage of cells with staining relative to the background (19). The evaluation of the extent of staining was based on the percentage of positive-stained cells among all the cells in the each case and scored from 0 to 4: 0, 0%, 1, 1–25%, 2, 26–50%, 3, 51–75% and 4, 76–100%. Similarly, the intensity of staining was based on the color of the certain cells in each case and scored from 0 to 3: 0, negative, 1, weak, 2, medium and 3, strong. The sum score of the intensity and extent of staining was used as the final score. Samples with a final score >2 were considered positive.

### Statistical analysis

All statistical analyses of results were performed using SPSS software system for Windows (version 13.0; SPSS, Inc., Chicago, IL, USA). The chi-squared (χ^2^) test was used to analyze the difference in the expression levels of ARTN and GFRα1 among different samples. Pearson’s correlation coefficient was calculated to evaluate the association between the expression of ARTN and GFRα1. Kaplan-Meier curves were produced to determine patient relapse-free survival (RFS) and overall survival (OS) rates. The statistical differences in survival among subgroups were compared using the log-rank test. P<0.05 was considered to indicate a statistically significant difference.

## Results

### Expression of ARTN and GFRα1 protein is upregulated in LSCC tissue samples

**Figure 1:**
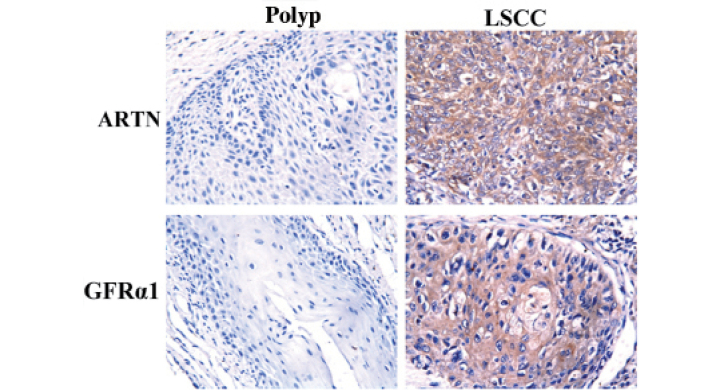
Expression of ARTN and GFRα1 in LSCC and polyp tissues specimens. Immunohistochemical analysis of ARTN and GFRα1 protein in LSCC and polyp. Low expression of ARTN and GFRα1 in polyp (left panels). High expression of ARTN and GFRα1 in LSCC (right panels). All images are counterstained with hematoxylin. Photomicrographs were captured at ×200 magnification. LSCC, laryngeal squamous cell carcinoma; ARTN, artemin; GFRα1, GDNF receptor α1.

Immunohistochemistry was used to determine the expression of immunoreactive protein for ARTN and GFRα1 in a cohort of specimens. Positive signals were observed in the cytoplasm of the squamous cell carcinoma cells or squamous epithelium of polyp tissues (Fig. 1). As shown in Table I, 53.9 and 51.3% of LSCC samples were positive for ARTN and GFRα1, respectively, whilst only 26.9% of normal squamous epithelium from patients with polyp were positive for ARTN and GFRα1 (P=0.015 and P=0.031, respectively).

### Correlation between the expression of ARTN and GFRα1 and clinicopathological features of LSCC

The association of tumor expression of ARTN and GFRα1 with the clinicopathological features of LSCC was then investigated. As observed in Table II, the expression of ARTN and GFRα1 was significantly associated with advanced pTNM stage (P=0.024 and P=0.006, respectively). However, no significant association was observed between the expression of ARTN and GFRα1 and any other clinicopathological characteristics, including tumor site, tumor differentiation and tumor lymph node metastasis (all P>0.05).

### Correlation between ARTN and GFRα1 expression and patient survival

**Figure 2:**
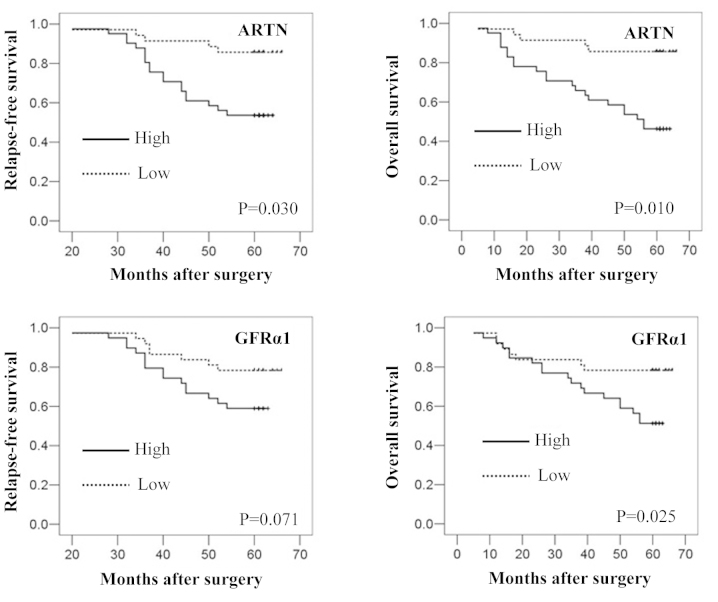
Correlation between ARTN and GFRα1 expression and patient survival. Kaplan-Meier analysis of the expression of ARTN and GFRα1 with the RFS and OS of patients with LSCC. LSCC, laryngeal squamous cell carcinoma; ARTN, artemin; GFRα1, GDNF receptor α1; RFS, relapse-free survival; OS, overall survival.

To determine the prognostic significance of ARTN and GFRα1 expression in patients with LSCC, Kaplan-Meier analyses were performed to correlate the expression of these proteins with the RFS and OS of patients. As observed in Fig. 2, patients with LSCC whose tumors were positive for expression of ARTN had a significantly lower five-year RFS or OS compared with patients whose tumors were negative for ARTN (P=0.030 and P=0.010, respectively). Similarly, expression of GFRα1 protein also predicted a significantly lower five-year OS compared with patients whose tumors were negative for GFRα1 (P=0.025). In addition, patients whose tumors expressed GFRα1 protein exhibited a lower RFS compared with patients whose tumors were negative for GFRα1 protein; however, this trend was not significant (P=0.071).

### Correlation between ARTN and GFRα1 expression

Correlation analysis was then conducted to determine the correlation between ARTN protein expression and the expression of GFRα1 protein in the same cohort of patients with LSCC. As was expected, Pearson’s correlation analysis revealed that the expression of ARTN was significantly correlated with the expression of GFRα1 in these patients (rs=0.527, P=0.001).

## Discussion

In this study, it was observed that a neurotrophic factor, ARTN, was expressed at significantly higher levels in LSCC compared with the levels in benign laryngeal polyp tissue samples. Furthermore, the expression of ARTN was demonstrated to be significantly associated with high tumor stage and poor survival. In addition, previous studies have suggested that ARTN has a role in the development and progression of diverse human carcinoma (9,10,12,20–22). In pancreatic ductal adenocarcinoma, ARTN has been reported to be highly expressed compared with normal pancreases, and stimulates the invasiveness of pancreatic cancer cells (13). Furthermore, the depletion of ARTN expression inhibits survival, invasion and anchorage-independent growth of both breast and endometrial cancer cells, while the forced expression of ARTN promotes these cellular behaviors (9,11). Molecularly, ARTN stimulates survival and anchorage-independent growth of human non-small cell lung cancer cells by upregulating BCL-2 expression (12). In addition, ARTN stimulates estrogen receptor-negative breast cancer cell growth, migration and metastasis by upregulating TWIST1 expression and activating the AKT pathway (21). These studies are in accordance with the results from the present study, suggesting an oncogenic role of ARTN in the progression of human malignancies.

ARTN has been reported to bind to and activate GFRα1 (5), a member of the GDNF receptor α family. In order to determine whether GFRα1 mediates the effects of ARTN in LSCC, the protein expression of GFRα1 in LSCC and matched normal tissues was analyzed, and the correlation between ARTN and clinicopathological features and patient survival outcome was investigated. The results from the present study revealed that the expression of GFRα1 was increased in cancerous tissues compared with the expression level in normal tissues and was also significantly associated with high tumor stage and poor survival of patients, which indicates that GFRα1 has a similar role to that of ARTN in LSCC. Furthermore, Pearson’s correlation analysis confirmed that the expression of GFRα1 has a significantly high correlation with ARTN expression. These results indicate that the functional effects of ARTN in the progression of LSCC may be mediated by GFRα1. Increased GFRα1 expression has been previously reported in breast cancer, and its expression is associated with tumor lymph node metastases and poor survival in patients (8,15). In addition, the stimulation of GFRα1-positive breast cancer cells with GDNF has been previously demonstrated to enhance cell proliferation and survival *in vivo* (8). In human neuroblastoma, Yoong *et al* demonstrated that GFRα1 promotes neurite outgrowth in tumor cells via the activation of ERK1/2, Rac1 and Cdc42 (23).

In conclusion, to the best of our knowledge, this study demonstrates for the first time the altered expression of ARTN and GFRα1 in LSCC and the association of the expression levels of these proteins with high stage disease and poor survival outcome for patients. The expression levels of ARTN and GFRα1 protein may therefore be useful as prognostic markers in LSCC. Whether other receptors of ARTN may also mediate its effects on the progression of LSCC remains to be determined.

## Figures and Tables

**Table I:** Expression of ARTN and GFRα1 in LSCC and polyp tissue specimens.

		Expression, n (%)	
		
Group	n	ARTN	GFRα1
LSCC	76	41 (53.9)^a^	39 (51.3)^b^
Polyp	26	7 (26.9)	7 (26.9)

a P=0.015 and

b P=0.031 vs. the polyp group.

LSCC, laryngeal squamous cell carcinoma; ARTN, artemin; GFRα1, GDNF receptor α1.

**Table II:** Association of ARTN and GFRα1 expression with clinicopathological parameters from patients with LSCC.

		ARTN		GFRα1	
			
Parameter	n	Expression, n (%)	P-value	Expression, n (%)	P-value
Age (years)					
≤60	40	19 (47.5)	0.235	17 (42.5)	0.105
>60	36	22 (61.1)		22 (61.1)	
Gender					
Male	70	39 (55.7)	0.291	37 (52.9)	0.358
Female	6	2 (33.3)		2 (33.3)	
Tumor site					
Supraglottic	22	14 (63.6)	0.521	15 (68.2)	0.131
Glottic	45	23 (51.1)		19 (42.2)	
Subglottic	9	4 (44.4)		5 (51.3)	
Tumor differentiation					
Well	28	17 (60.7)	0.122	16 (57.1)	0.681
Moderate	32	19 (59.4)		16 (50.0)	
Poor	16	5 (31.3)		7 (43.8)	
pTNM stage					
I–II	35	14 (40.0)	0.024^a^	12 (34.3)	0.006^a^
III–IV	41	27 (65.9)		27 (65.9)	
Lymph node metastasis					
Yes	26	13 (50.0)	0.619	11 (42.3)	0.257
No	50	28 (56.0)		28 (56.0)	

a P<0.05.

LSCC, laryngeal squamous cell carcinoma; ARTN, artemin; GFRα1, GDNF receptor α1.
